# *FOXP2* variation in great ape populations offers insight into the evolution of communication skills

**DOI:** 10.1038/s41598-017-16844-x

**Published:** 2017-12-04

**Authors:** Nicky Staes, Chet C. Sherwood, Katharine Wright, Marc de Manuel, Elaine E. Guevara, Tomas Marques-Bonet, Michael Krützen, Michael Massiah, William D. Hopkins, John J. Ely, Brenda J. Bradley

**Affiliations:** 1 Center for the Advanced Study of Human Paleobiology, Department of Anthropology, The George Washington University, 800 22nd Street NW, Suite 6000, Washington, DC 20052 USA; 2 Department of Chemistry and Center of Biomolecular Science, The George Washington University, 800 22nd Street NW, Washington, DC 20052 USA; 3 Institute of Evolutionary Biology (UPF-CSIC), PRBB, Dr Aiguader 88, 08003 Barcelona, Spain; 4 Catalan Institute of Research and Advanced Studies (ICREA), Passeig de LIuis Companys 23, 08010 Barcelona, Spain; 5 CNAG-CRG, Centre for Genomic Regulation (CRG), Barcelona Institute of Science and Technology (BIST), Baldiri I Reixac 4, 08028 Barcelona, Spain; 6 Evolutionary Genetics Group, Department of Anthropology, University of Zurich, Winterthurerstrasse 190, CH-8057 Zurich, Switzerland; 7 Neuroscience Institute, Georgia State University, 33 Gilmer Street SE, Atlanta, GA 30322 USA; 8 Division of Developmental and Cognitive Neuroscience, Yerkes National Primate Research Center, 201 Dowman Drive, Atlanta, GA 30322 USA; 9 MAEBIOS, 1610 Juniper Drive, Alamogordo, NM 88310 USA; 10 Department of Anthropology, Yale University, 10 Sachem Street, New Haven, CT 06511 USA

## Abstract

The gene coding for the forkhead box protein P2 (*FOXP2*) is associated with human language disorders. Evolutionary changes in this gene are hypothesized to have contributed to the emergence of speech and language in the human lineage. Although *FOXP2* is highly conserved across most mammals, humans differ at two functional amino acid substitutions from chimpanzees, bonobos and gorillas, with an additional fixed substitution found in orangutans. However, *FOXP2* has been characterized in only a small number of apes and no publication to date has examined the degree of natural variation in large samples of unrelated great apes. Here, we analyzed the genetic variation in the *FOXP2* coding sequence in 63 chimpanzees, 11 bonobos, 48 gorillas, 37 orangutans and 2 gibbons and observed undescribed variation in great apes. We identified two variable polyglutamine microsatellites in chimpanzees and orangutans and found three nonsynonymous single nucleotide polymorphisms, one in chimpanzees, one in gorillas and one in orangutans with derived allele frequencies of 0.01, 0.26 and 0.29, respectively. Structural and functional protein modeling indicate a biochemical effect of the substitution in orangutans, and because of its presence solely in the Sumatran orangutan species, the mutation may be associated with reported population differences in vocalizations.

## Introduction

Language is a defining feature of human uniqueness. Therefore, the cognitive, motor, and neural foundations that distinguish human speech and language from other animal communication systems have been a central focus of research in the social and biological sciences for more than 200 years^1,2^. To address the puzzle of human language origins, it is essential to examine the cognitive processes, neurobiology, and genetics underlying this unique form of communication in an evolutionary context, particularly in comparison to our species’ closest living relatives, the great apes (chimpanzees, bonobos, gorillas and orangutans)^3^.

Although it has been presumed that great apes are limited in their vocal capacity to produce the range of sounds in human speech^4,5^, they do show local variation in vocal calls that appear to be inherited across generations through social transmission (for review see^6^). Such vocal learning refers to the ability of an individual to modify the acoustic features or timing of existing species-typical calls, or to learn new calls altogether, expanding the vocal repertoire. Evidence for vocal learning by call modification has been documented in several primate species and recent studies also report some capacity for vocal invention, which typically encompasses voiceless vocalizations^6–11^. For example, in chimpanzees, the use of novel vocal signals, such as attention-getting sounds^7,9^ has been reported in captive populations, as a means of attracting the attention of an otherwise inattentive audience^12^. The capacity to produce these vocalizations is heritable and possibly socially learned, suggesting that these apes have voluntary control of both vocal signals and orofacial musculature^8^. Some apes can also acquire and use symbols during two-way interspecies communication through alternative and augmentative systems such as American Sign Language^13^ and visual-graphic symbols^14,15^. Furthermore, many parallels have been found in the gestures of apes compared to preverbal children, such as initiation and responding to pointing cues, intentional and referential signaling, and the elaboration and repair of failed communication^16–18^. Many nonverbal behaviors found in great apes, such as joint-attention^19^, have also been observed in preverbal children just prior to the onset of speech, which might serve as part of the cognitive foundation of language development^20^. Thus, in terms of understanding language evolution, great apes represent key reference species.

The genetic changes responsible for the human capacity for increased vocal learning likely occurred since our lineage split from chimpanzees and bonobos. One well-studied candidate is the gene coding for the transcription factor *FOXP2* (forkhead box P2)^21,22^. *FOXP2* is the first gene that was discovered to be associated with language disorders and fine orofacial motor control, as two functional copies are required for normal development of speech and language in humans^21–23^. Mutations affect primarily the coordination of orofacial movements required for speech^24^. Several recent studies compared the evolution of this gene in primates and other species^21,25–29^. Interestingly, the protein coding sequence is among the most highly conserved 5% of proteins in vertebrates, and its role in regulating vocal learning and communication appears to be shared across a range of animal species^21,29–32^, as is the expression of the gene in several key brain regions related to language and fine motor control^28,33,34^. More specifically, the gene is crucial for the development and function of brain circuits involving the neocortex, basal ganglia and cerebellum^32,35–37^. *FOXP2* mRNA is expressed in these brain regions among mammals and avians, reinforcing the view that it plays a role in speech in humans and motor learning in other species, such as birds and mice^28,38^.

Although a number of nucleotide changes have accumulated in *FOXP2* since primates diverged from the mouse lineage around 70 million years ago, only one of these changes resulted in an amino acid substitution (i.e. a nonsynonymous mutation)^27^. Strikingly, two more amino acid differences are found specifically on the human lineage, with an additional fixed lineage-specific difference in orangutans^27^. This indicates that modern humans have a uniquely derived version of *FOXP2* that arose since the last common ancestor shared with the *Pan* lineage, only 4 to 6 million years ago^3,27^. In comparison to the highly conserved sequence, the rate of amino acid substitutions in this gene in modern humans is higher than expected by chance, indicating a signal of accelerated evolution^27^. Subsequent functional assessments of the human-specific changes to *FOXP2* using mice engineered to express the human variant of the gene revealed changes in synaptic plasticity, axon and dendrite outgrowth, and physiological activity in medium spiny neurons of the striatum, supporting the idea that the human variant of *FOXP2* causes alteration in brain development^36,39^. To date, however, it remains unclear when in the past these amino acid substitutions first occurred, as modern humans share them with both Neanderthals and Denisovans, indicating they originated at least ~400,000 years ago^40^. Recent findings suggest that other regulatory changes in the gene unique to modern humans lie at the base of the selection signal^40^, and experimental evidence shows that the human FOXP2 variant differentially regulates downstream targets compared to the ancestral version found in chimpanzees^41^.

Despite such tremendous interest in the *FOXP2* gene among linguists, anthropologists, geneticists and neuroscientists, our understanding of within-species variation of this gene among nonhuman primates remains limited (but see^42,43^). Interestingly, the gene has been better studied in bats than primates, and despite the high conservation of the protein sequence in a majority of mammals, bats show remarkable coding variation in the *FOXP2* gene which may be linked to differences in echolocation systems among species^26^. Thus, further examination of *FOXP2* variation across primate species holds the potential to provide insight into the evolution of vocal control and communication systems within the human lineage.

The aim of this study was to assess *FOXP2* coding variation in a relatively large sample of great apes. We also investigated potential within-species allelic length variation in the two polyglutamine (poly Q) tracts located in exons 5 and 6 of the gene. Poly Q tracts are encoded by a mixture of CAG and CAA codons repeated in tandem; these types of repeats typically have higher mutation rates and can serve as a functional modulator of eukaryotic transcription factors^44^. Poly Q tract variation has been shown to impact gene expression by regulating gradients of expression akin to a “tuning-knob” effect, as shown for example with the *RUNX*2 transcription factor^45^. As *FOXP2* both upregulates and downregulates a large array of different target genes in human basal ganglia and inferior frontal cortex^41^, poly Q length variation could have important consequences for gene expression. Despite the nature of these repeats, length variation in the gene in humans is rare^23,46^ and is therefore commonly overlooked. Studies in nonhuman primates, in contrast, do report between-species length differences of these repeats^27,43^, but none of these studies to date have investigated within-species length variation of these poly Q tracts.

## Results

### *FOXP2* coding variation

**Figure 1:**
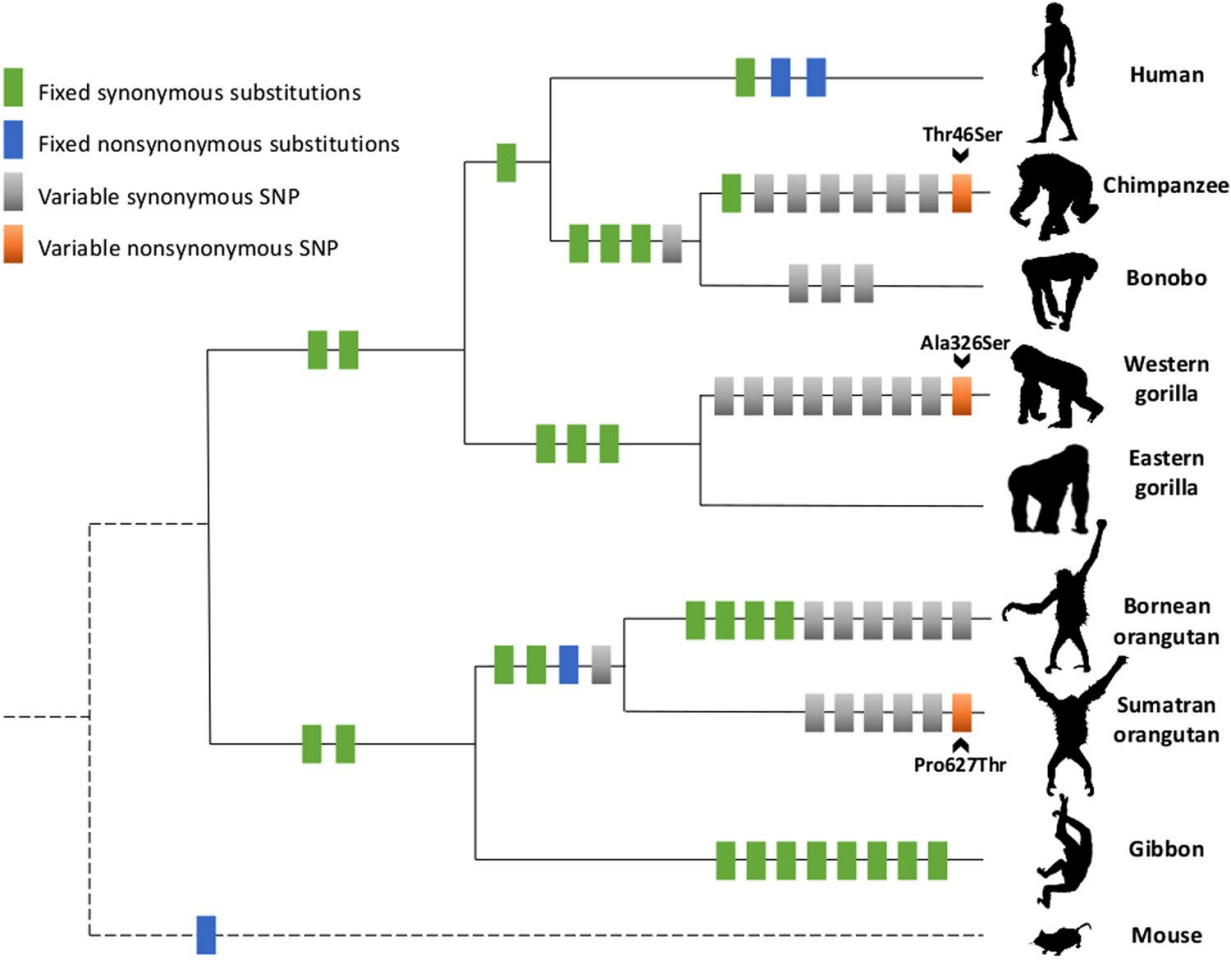
Single nucleotide variation in the coding region of *FOXP2* across apes. Amino acid polymorphisms are indicated by name and location of the substitution in the amino acid sequence.

**Figure 2:**
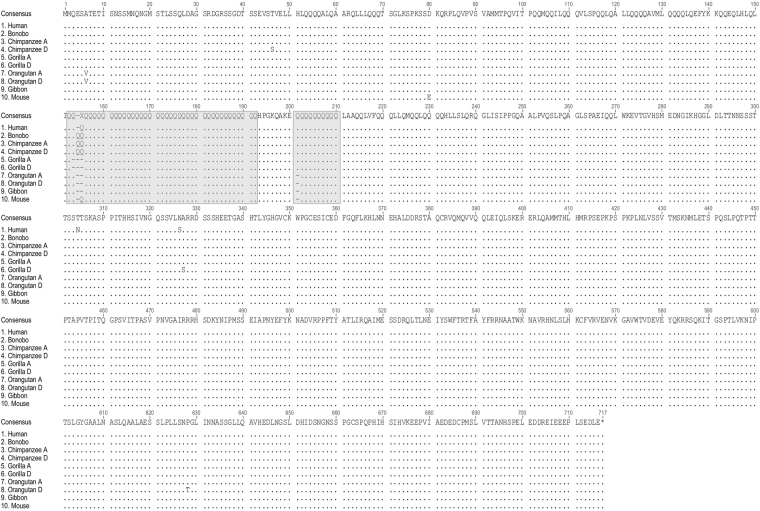
Alignment of the FOXP2 amino-acid sequences across apes. Both polyglutamine stretches (glutamine, denoted as Q, repeated 39–42 and 9–11 times in tandem see positions 151–210) are shaded. Dots indicate similarity to the consensus sequence. For species where within-species variation in amino acid substitutions were found, an individual with the ancestral (**A**) and derived (**D**) sequence are shown.

We identified 52 single nucleotide variants (SNV) among apes, of which 21 were fixed species-specific substitutions, and 31 were within-species single nucleotide polymorphisms (SNPs) (Fig. 1, Table S5). Out of 31 within-species SNPs found, three were nonsynonymous substitutions (Fig. 2). In chimpanzees, an A/T SNP in the first translated exon resulted in a Threonine to Serine substitution (Thr46Ser), present in just one individual in our sample (minor allele frequency = 0.008). In gorillas, a G/T SNP in exon 7 leads to an Alanine to Serine substitution (Ala326Ser). This SNP is only found in western lowland gorillas (*Gorilla gorilla gorilla*), with the ancestral G allele present at a higher frequency (0.74). Both allele and genotype frequencies were in Hardy Weinberg equilibrium (*X* ^2^ = 1.05, df = 1, p = 0.305), indicating that potential genotyping errors such as allelic dropout, did not pose a problem in this study. In orangutans, a C/A SNP in exon 16 causes a Proline to Threonine substitution (Pro626Thr). The latter SNP is found only in Sumatran orangutans (*Pongo abelii*), with the ancestral C allele again present at a higher frequency (0.71). Allele and genotype frequencies were in Hardy Weinberg equilibrium (*X* ^2^ = 2.20, df = 1, p = 0.138).

We also identified length variation in the poly Q tracts of both orangutans and chimpanzees (Table 1). In orangutans, two alleles were found for each poly Q tract, always differing in length by one 3 bp glutamine (Q) codon. In chimpanzees, two alleles were found for poly Q tract one (exon 5) and three alleles were distinguished in poly Q tract two (exon 6), again all differing by one Q codon. Allele and genotype frequencies were in Hardy Weinberg equilibrium for both loci in chimpanzees (Q1: *X* ^2^ = 0.47, df = 1, p = 0.491; Q2: *X* ^2^ = 0.50, df = 3, p = 0.919), and for Q2, but not Q1 in orangutans (Q1: *X* ^2^ = 14.88, df = 1, p = 0.0001; Q2: *X* ^2^ = 0.04, df = 1, p = 0.850).

**Table 1:** Frequency and percentage of poly Q alleles found in Sumatran orangutans (N = 32) and chimpanzees (N = 54).

	Allele (bp)	#Q	Sumatran orangutan		Chimpanzee	
			Frequency	Percentage	Frequency	Percentage
Poly Q1	163	39	62	96.9	0	0
	166	40	2	3.1	0	0
	169	41	0	0	101	93.5
	172	42	0	0	7	6.5
Poly Q2	84	9	50	78.1	7	6.5
	87	10	14	21.9	98	90.7
	90	11	0	0	3	2.8

### Prediction of functional consequences and protein structure modeling for amino acid substitutions

**Figure 3:**
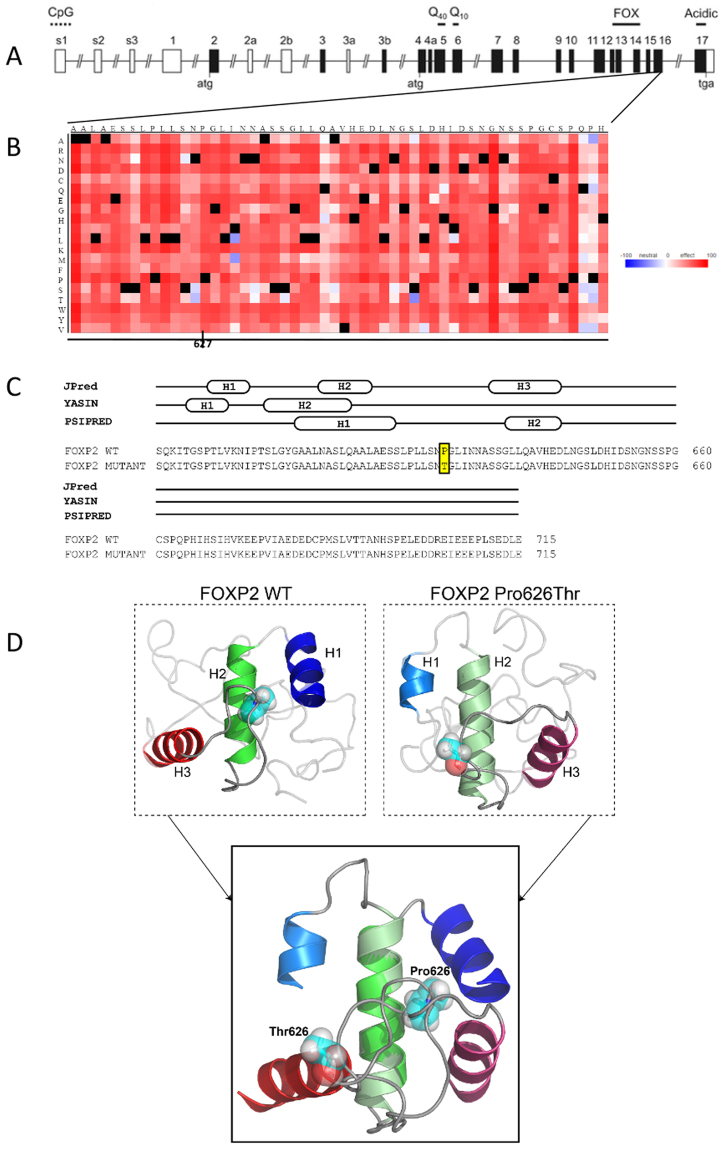
(**A**) Schematic representation of the *FOXP2* gene. Boxes represent exons and lines represent introns. Translated exons included in this study are shaded in black. The domains coded for by the exons are shown above: two polyglutamine tracts (Q40 and Q10), a zinc-finger motif (ZnF), a leucine-zipper (LeuZ), the forkhead domain FOX, and an acidic C-terminus. CpG marks the site of a CpG island. (**B**) Heatmap showing predicted functional consequences of P626T mutation found in orangutans. The stronger the predicted effect, the redder; the stronger the predicted neutrality, the bluer. (**C**) Sequence alignment of the wild-type and Pro626Thr mutant FOXP2-CTR proteins. Secondary structure predictions are shown for each of the three algorithms used. (**D**) Comparison of the predicted tertiary structures of the wild-type and Pro626Thr FOXP2-CTR proteins; helix 2 of each structure are superimposed for comparison. Residues Pro626 and Thr626 are shown in cyan and as both sticks and spheres.

SNAP2 prediction of the functional consequences of the chimpanzee Thr46Ser, gorilla Ala326Ser and orangutan Pro626Thr substitutions resulted in SNAP2 effect scores of −65 (expected accuracy 82%), −76 (expected accuracy 87%) and 64 (expected accuracy 80%), respectively (Fig. 3B).

Next, the secondary and tertiary protein structural differences for ancestral (wild-type) and species-specific (mutant) FOXP2 regions containing the Ala326Ser and Pro626Thr substitutions were predicted. We refrained from structural modeling for Thr46Ser, because the mutation was only present in one chimpanzee and functional modeling predicted a neutral effect. The Ala326Ser and Pro626Thr substitutions were located in the region following the N-terminal Poly Q region (FOXP2-APQ, residues 210–339), and the FOXP2 C-terminal region (FOXP2-CTR, residues 583–715), respectively. Secondary structure prediction of both regions showed similar results when modeled as full-length *FOXP2* sequences or as isolated regions. For both regions, two of the secondary prediction algorithms predicted two α-helical segments, while the other algorithm identified three. The helical segments were identified primarily within the first two-thirds of the region with the last 50 C-terminal amino acids predicted to be mainly unstructured. No differences in the secondary structure predictions were observed when the sequences included the Ala326Ser and Pro626Thr mutations. Both mutation sites were observed in regions predicted to be unstructured loops (Fig. 3C and Supplementary Fig. S1A). The Ala326Ser mutation is located in the last ~50 unstructured amino acids of the FOXP2-APQ region. For comparison, the secondary structure of the FOXP2 forkhead domain matched well with its known structure, consisting of four α-helices and two β-strands.

Tertiary structure predictions of the wild-type and Ala326Ser FOXP2-APQ showed that the region consists of three α-helices, one of which adopts a long central helix (Supplementary Fig. S1B). The α-helices encompass residues Ala210-Gln239, Pro262-Val272 and Met278-Gly286. As expected, Ala326 is in an unstructured region approximately 15 residues from the C-terminus. Analyses of all ten predicted structures of wild-type FOXP2-APQ revealed variation in the orientations of α-helices 2 and 3 and the location of the Ala326 residue. To identify the effect of the Ala326Ser mutation, the first and longest helix between the two sets of structures were superimposed. The structural comparison revealed variation in the orientations and locations of the Ser326 residue and α-helices 2 and 3, which were similar to the variation observed in the predicted structures of the wild-type FOXP2-APQ (Supplementary Fig. S1B). It is thus unclear whether the Ala326Ser mutation has a structural impact within this region. We then investigated the S^321^SVLNXRRDS region, in which X indicates the mutation (A or S), for phosphorylation signatures since serine amino acids are targeted by kinases. When X is alanine (wild-type), Ser322 and Ser330 would be sites for G-protein coupled receptor (GPCR) kinase and protein kinase C, respectively. When X is serine (mutation), the Ser326 introduces motifs for MAP kinases (SxxxpS^326^), PKA (S^326^RR), pyruvate dehydrogenase kinase (pS^326^xxDxx), glycogen synthase kinase (S^322^xxxpS^326^), and casein kinase (S^326^RRD), in which x = any amino acid (Supplementary Fig. S1C).

Next, we examined the Pro626Thr mutation within the FOXP2-CTR region. Given that Pro626 is observed in a loop region between α-helices 2 and 3, it is unclear how the Pro626Thr mutation might affect the structure and function of FOXP2. The predicted structures of the wild-type and Pro626Thr FOXP2-CTR contain three α-helices, found within the N-terminal half of the protein, while the C-terminal half consists of unstructured loops (Fig. 3D). The three α-helices encompass residues Pro591-Leu601, Gly604-Ala616 and Ala639-D649, respectively, held together by hydrophobic interaction. In contrast, the α-helices for the mutant FOXP2-CTR are 1–3 amino acids shorter. To identify the effect of the Pro626Thr mutation located in the loop between α-helices 2 and 3, the central and longest helix between the two structures was superimposed to reveal that α-helices 1 and 3 are oriented differently with respect to helix 2 (Fig. 3D). Likewise, the positions of Pro626 and Thr626, in their respective structures, are different.

Models of FOXP2 protein evolution (dN/dS sites analysis) show evidence of strong purifying selection across the polypeptide (Supplementary Fig. S2). Neither SLR (sitewise likelihood-ratio) nor codeml analyses detected evidence of positively selected sites, and likelihood ratio tests using both methods indicated a model of predominantly purifying selection with some sites experiencing neutral selection to be the most likely model. The three sites where we identified nonsynonymous single nucleotide polymorphisms had low dN/dS, with Thr46Ser and Pro626Ser yielding ratios of 0, and Ala326Ser yielding a ratio of 0.26.

## Discussion

Within-species coding sequence variation of *FOXP2* in the largest samples of great apes analyzed to date reveals likely functional variation potentially associated with communication skill and orofacial motor control. Length variation was found in both polyglutamine tracts in chimpanzees and orangutans. Additionally, three species-specific nonsynonymous SNPs were found, Thr46Ser in chimpanzees, Ala326Ser in gorillas and Pro626Thr in orangutans. Protein structure modeling did not indicate a clear effect for Ala326Ser, but it did reveal a potential impact of the Pro626Thr variant in orangutans: the mutation was predicted to alter the tertiary structure of a DNA binding interface in the C terminal region. Our results further confirmed interspecies differences in functional coding sequences between humans and great apes, as reported previously^27,42,43^.

The between-species comparison of the length of the two poly Q tracts is generally congruent with earlier reports. However, chimpanzees and orangutans show within-species variation in the length of both poly Q tracts, which is surprising given that reported variation in these tracts is rare in human studies^46–49^. One explanation is that orangutans and chimpanzees have much higher genetic diversity than humans, with populations having diverged millions of years ago^3^. Poly Q tract variation in transcription factors can potentially impact expression of downstream genes via a sort of tuning-knob effect^50^. Notably, the few studies that do report variation in these poly Q tracts show that they are likely functional, as higher frequencies of rare alleles are found in human individuals suffering from speech and/or language impairments^46,48,49^. Since the sequencing coverage at the position of these poly Q tracts was low in the whole-genome data, and we had an insufficient number of DNA samples for a large enough set of unrelated gorillas and bonobos, we were only able to examine poly Q length variation in orangutans and chimpanzees. Further sampling is required for the other species to determine whether potential within-species length variation exist in these tracts. Interestingly, chimpanzees show remarkable individual variation in the frequency and consistency of the use of vocal attention-getting sounds. The source of individual variation in the use of such vocalizations remains largely unknown, but since it is heritable^8^, it likely has a genetic component. Therefore, the individual genetic variation in both poly Q tracts presented in this study offers promising candidate loci for future research.

The Thr46Ser mutation identified in chimpanzees was present in just one individual, indicating that it is a rare variant. Therefore, it is difficult to investigate its potential phenotypic significance. Because of this, and the fact that functional SNAP2 prediction resulted in a likely neutral effect, we did not perform further structural modeling for this variant. In humans, many cases of rare de novo and familial nonsynonymous mutations and deletions in *FOXP2* have been reported, and disruption of the gene typically results in severe motor speech disorders, or differences in cognitive and/or generalized motor skills^22^ but see^51^. It is unclear whether the Thr46Ser mutation affects this individual chimpanzee’s vocal, cognitive or motor skills, and it could potentially be due to a sequencing error. Selection modeling shows that the dN/dS ratio found at Ala326Ser was slightly higher than in other sites of the sequence, suggesting selection at this site may be somewhat more relaxed.

The second and third species-specific nonsynonymous variants, Ala326Ser and Pro626Thr, are present in western lowland gorillas and Sumatran orangutans, respectively. Both mutations had a relatively high frequency in the populations in which they were present, occurring in almost half of the genotyped individuals. Based on the three-dimensional homology search algorithm, DALI, the spatial orientation of the three α-helices in both the FOXP2-APQ and FOXP2-CTR structures revealed a helix-turn-helix motif commonly observed in DNA binding proteins^52^, such as transcription factors and repressor proteins. This is consistent with both regions located adjacent to the DNA-binding forkhead domain. The gorilla Ala326Ser variant is located in exon 7, which is the same exon that contains both previously reported fixed human nonsynonymous mutations^27^. The orangutan Pro626Thr mutation is located in exon 16, close to exons 12–14, which code for the DNA binding forkhead domain.

Although FOXP2 is under strong purifying selection and no sites showed significance evidence of directional selection, there are notable patterns of potential convergence across mammals. The exons of interest where variation was found in apes (exon 7, 16 and 17) also have the highest numbers of nonsynonymous fixed substitutions in bats^26^. This is interesting given that previous research suggests that the bat variants in these regions likely have adaptive significance, as opposed to being due to relaxed selection^26^.

For the Ala326Ser mutation found in gorillas, both functional and structural protein modeling revealed no clear differences between the wild-type and mutant FOXP-APQ region. However, since the mutation introduces a serine at position 326, and serine amino acids are commonly targeted by kinases, the mutation may still alter phosphorylation signatures at this position. The serine introduces more possibilities for phosphorylation by additional kinases that can affect how FOXP2 interacts with other proteins associated with gene expression. Although post-transcriptional modifications including sumoylation can have a functional impact on FOXP2^53^, there is no empirical evidence of phosphorylation of the ancestral version of FOXP2 (but see^27^). While the Ala326Ser mutation may increase the chance of phosphorylation, it is unclear if this is actually the case at Ser326, so further experimental assays are needed to investigate the functionality of this SNP in gorillas. However, little evidence from field studies indicates differences in vocal repertoire, vocal learning or orofacial motor control, either within western lowland gorillas or between gorilla populations that could be attributable to the SNP found in this study^54^. Although there are reported differences in the production of “raspberry” vocalizations in wild mountain gorillas, but not in western lowland gorillas^54^, this observation is not easily reconciled with our finding that the SNP is present in some but not all western gorillas. Future studies of wild gorilla vocal behavior, nevertheless, might reveal if there is a relationship between this phenotypic variation and the genetic polymorphism we describe here.

For the Pro626Thr mutation found in orangutans, both functional and structural protein modeling showed a high likelihood that the mutation alters the native protein. Interestingly, compared to FOXP2-APQ, the FOXP2-CTR region does not contain a significant amount of lysine, arginine and aromatic amino acids, commonly involved in protein-nucleic acid interactions. Instead, it consists of many polar residues (serine, glutamine, asparagine, threonine) at the helix-turn-helix DNA binding interface that can form hydrogen bonds with the bases of DNA. The structure of the Pro626Thr mutant showed a complete rearrangement of these polar residues that may affect the DNA binding property of FOXP2-CTR. Further experimental evidence remains to be collected to confirm the exact functional effect of this mutation. However, it is interesting to note that the Pro626Thr mutation was found solely in Sumatran orangutans, which show remarkable differences in behavior and vocal skills compared to their Bornean sister species.

The Sumatran orangutans live in habitats with more stable food availability, they are more sociable and show lower frequencies of forced mating^55^. Notably, these populations also differ in their vocal repertoire and the pitch frequency of vocalizations^56,57^. For example, male long calls differ consistently between Sumatran and Bornean orangutans in number of pulses per call, call speed, call duration, bandwidth, pulse duration and dominant frequency^57^. Furthermore, male orangutans in Borneo are reportedly larger than Sumatran males and are therefore expected to have lower call pitches, yet exactly the opposite is found^57^. Since *FOXP2* variation has been linked to differences in vocal learning and vocalization frequency differences in a variety of species^28,32,38^, the Pro626Thr mutation could be associated with reported vocal differences, not only between the two orangutan species, but also between individuals belonging to different populations within Sumatra, since the SNP is not present in all Sumatran individuals^57^. Results from captive studies also show that orangutans have the ability for vocal fold control^10^ and that they can more skillfully imitate human speech than any other apes^11^. This combined with evidence suggesting that Sumatran individuals are more avid oral tool users compared to the Bornean orangutans^58^, may be related to reported associations between *FOXP2* mutations and levels of orofacial motor control^25^. These observations suggest that future studies of Pro626Thr may reveal salient individual or population differences in vocal behavior in Sumatran orangutans, although we cannot entirely rule out demographic history of these species as an additional factor influencing the genotype distribution pattern in this study^59^.

Overall, despite the relatively large number of great apes sampled in this study, the number of nonsynonymous substitutions found was low, indicating that *FOXP2* is highly constrained and likely under purifying selection in great apes, and mammals in general^26,27^. Therefore, investigation of the impact of rare nonsynonymous variants found here in great apes could shed light on the proximate mechanisms shaping individual, population or even species level differences in vocal skills and orofacial motor control. Furthermore, although this study focused on coding variation because of its direct impact on protein structure and function, variation in regulatory and intronic regions, as well as tissue-specific alternative splice variants, also warrant further study^47,60^.

## Methods

### Genome sequencing, assembly and annotation

Genotyping was performed using publicly available whole genome data for chimpanzees (*Pan troglodytes troglodytes* N = 18, *Pan troglodytes verus* N = 12, *Pan troglodytes schweinfurthii* N = 16, *Pan troglodytes ellioti* N = 10, *Pan troglodytes* unknown subspecies N = 3), bonobos (*Pan paniscus*, N = 9), gorillas (*Gorilla beringei beringei* N = 7, *Gorilla beringei graugeri* N = 9, *Gorilla gorilla diehl* N = 1, *Gorilla gorilla gorilla* N = 27) and orangutans (*Pongo pygmaeus* N = 20, *Pongo abelii* N = 17)^3,61,62^. All genomes were mapped to human genome (version hg19) using BWA-MEM v0.7.5a-r405 (http://bio-bwa.sourceforge.net/bwa.shtml) with default parameters. After removing duplicates using PICARD v1.91 (https://sourceforge.net/projects/picard/files/picard-tools/1.91/), single nucleotide polymorphisms were called using GATK *UnifiedGenotyper* (https://software.broadinstitute.org/gatk/documentation/tooldocs/current/org_broadinstitute_gatk_tools_walkers_genotyper_UnifiedGenotyper.php). Gene consensus sequences were built for each individual using *vcfconsensus* (http://vcftools.sourceforge.net/man_latest.html). Intronic regions and non-protein coding exons were removed from the sequences and remaining exons were aligned to the human *FOXP2* coding reference sequence (Ensembl: ENSG00000128573) using Geneious (version 6.0.6).

### Sanger sequencing of exons

To maximize our sample size for this study, we included additional data from a preliminary study^42,43^, where genomic DNA was extracted from peripheral whole blood of four African-born chimpanzees (*Pan troglodytes*, including 1 *P.t.verus*, 1 *P.t.troglodytes*, 1 *P.t.schweinfurthii*, and 1 probable P*.t.troglodytes/schweinfurthii*), bonobos (*Pan paniscus*, N = 2), western lowland gorillas (*Gorilla gorilla gorilla*, N = 2), Sumatran orangutans (*Pongo abelii*, N = 2) and white-handed gibbons (*Hylobates lar*, N = 2). For details about the chimpanzees, subspecies ascertainment, DNA extraction, polymerase chain reaction (PCR) and DNA sequencing, see^63^. All coding exons (Fig. 3A) were amplified by PCR (primers and PCR conditions shown in Supplementary Table S1) and Sanger sequenced on a Li-cor 4200 DNA sequencer analyzer following manufacturer’s specifications. Multiple alignments of trimmed nucleotide and deduced amino acid sequences for all exons for each species were performed using Geneious (version 6.0.6). Resulting sequences are deposited in NCBI under accession numbers MG547712-MG547713, MG547714, MG547715, MG547716, MG547717, MG547718, MG547719, MG547720 and MG547721.

### Microsatellite analysis of polyglutamine tracts

Since the sequencing coverage at the position of the poly Q tracts was low in the whole-genome data, we scored microsatellite genotypes for both tracts in available panels of chimpanzees (N = 54) and Sumatran orangutans (N = 32). For primer design, PCR conditions and fragment length analyses see supplementary information (Table S3). Individuals were genotyped using automated capillary electrophoresis on Applied Biosystems Genetic Analyzer platforms (DNA Analysis Facility at Yale University).

### Function and structure prediction of coding variants

To infer the putative functional consequences of the identified coding variants, we first used SNAP2 to predict the effect of variants on protein function^64^. SNAP2 is a trained classifier that is based on a machine-learning device called “neural network”. It distinguishes between effect and neutral variants/non-synonymous SNPs by taking a variety of sequence and variant features into account. The effect of a variant is believed to be of importance to the native protein function if the SNAP2 score exceeds 50, neutral if the score is below −50 and unreliable when between 50 and −50.

The secondary structures of the full-length FOXP2 (residues 1–715) and the C-terminal region (residues 583–715, FOXP2-CTR) that follows the forkhead domain of the wild-type FOXP2 protein were predicted by PSIPRED^65^, Jpred^66^, and YASPIN^67^. A third prediction was performed for residues 210–339, that follows the N-terminal Poly Q region of the protein (FOXP2-APQ). The secondary structures of the FOXP2-APQ and FOXP2-CTR regions containing the Ala326Ser and Pro626Thr mutations were also predicted, respectively. As a control, the sequence encompassing the forkhead domain (residues 503–582), which has a known structure, was also submitted for secondary structure prediction.

The tertiary structures of the wild-type and mutant FOXP2-APQ and FOXP2-CTR were predicted using the *ab initio* modeling program QUARK^68^. While numerous *ab initio* modeling programs are available, QUARK has proven to be more accurate when predicting the structure of helical proteins^68^. For each of the wild-type and both mutant proteins, ten structures were predicted. Structures were ranked by their template modeling (TM) scores, the highest of which corresponds to the best structure, represented by the first model (Model 1). We also submitted two protein sequences for which the structures are known, including the forkhead domain of FOXP2. For these two proteins, the overall predicted and experimental structures have similar folds with root-mean-square deviation values of backbone atom superposition of 4.567 (forkhead, PDB accession code: 2A07) and 4.327 (UBR5 PABC domain, PDB accession code: 3NTW). In case the predicted structures of the wild-type and mutant FOXP2 regions did not yield apparent differences, further investigation was done to identify potential changes in phosphorylation motifs that could affect the function of the region using NetPhorest^69^.

To further identify potential functional implications of variants, we also investigated the evidence of selection on amino acid sites within FOXP2. We examined site-specific dN/dS ratios across a large alignment of 57 placental mammals (Supplementary Table S4), including 12 primates and five bats. A very low dN/dS ratio (i.e. close to zero) suggests that a site has been evolutionarily highly conserved, and thus is likely critical to protein structure/function. For details on dN/dS ratio calculations see Supplementary Methods 1.

### Data Availability

The sequences generated during the current study are available at the NCBI repository.

### Ethical statement

No animals were sacrificed or sedated for the purpose of this study. All aspects of this research adhered to the American Psychological Associations guidelines for the ethical treatment of animals in research.

## Electronic supplementary material

Supplementary information
