# Supplementary material for: FOXP2 variation in great ape populations offers insight into the evolution of communication skills
Source: Sci Rep. 2017 Dec 4;7:16866. doi: 10.1038/s41598-017-16844-x (PMC5715162; doi:10.1038/s41598-017-16844-x)

Supplementary information

FOXP2 variation in great ape populations offers insight into the evolution of communication skills

Nicky Staes[1,*], Chet C Sherwood[1], Katharine Wright[2], Marc de Manuel[3], Elaine E Guevara[1], Tomas Marques-Bonet[3,4,5], Michael Krützen[6], Michael Massiah[2], William D Hopkins[7,8] John J Ely[9] and Brenda J Bradley[1]

[1] Center for the Advanced Study of Human Paleobiology, Department of Anthropology, The George Washington University, 800 22nd Street NW, Suite 6000, Washington, DC, 20052, USA

[2] Department of Chemistry and Center of Biomolecular Science, The George Washington University, 800 22nd Street NW, Washington, DC, 20052, USA

[3] Institute of Evolutionary Biology (UPF-CSIC), PRBB, Dr Aiguader 88, 08003 Barcelona, Spain

[4] Catalan Institute of Research and Advanced Studies (ICREA), Passeig de Lluis Companys 23, 08010, Barcelona, Spain

[5] CNAG-CRG, Centre for Genomic Regulation (CRG), Barcelona Institute of Science and Technology (BIST), Baldiri I Reixac 4, 08028 Barcelona, Spain

[6] Evolutionary Genetics Group, Department of Anthropology, University of Zurich, Winterthurerstrasse 190, CH-8057 Zurich, Switzerland

[7] Neuroscience Institute, Georgia State University, 33 Gilmer Street SE, Atlanta, GA, 30322, USA

[8] Division of Developmental and Cognitive Neuroscience, Yerkes National Primate Research Center, 201 Dowman Drive, Atlanta, GA, 30322, USA

[9] MAEBIOS, 1610 Juniper Drive, Alamogordo, NM, 88310 USA

* Corresponding author: nstaes@gwu.edu

Table S1: FOXP2 coding exon coordinates in GRCh37/ hg19 as used for numbering in this study

		Full exon			Translated region of exon		
Exon nr	Ensembl nr	Start	End	Length (bp)	Start	End	Length (bp)
Exon 2	ENSE00003592860	114426502	114426679	178	114066567	114066734	167
Exon 3	ENSE00003598938	114534617	114534706	90	114174672	114174761	89
Exon 4	ENSE00003561477	114628540	114628677	138	114268595	114268732	137
Exon 5	ENSE00003784625	114629856	114630005	150	114269911	114270060	149
Exon 6	ENSE00003689434	114631528	114631705	178	114271583	114271760	177
Exon 7	ENSE00003599193	114642410	114642623	214	114282465	114282678	213
Exon 8	ENSE00003569605	114644685	114644789	105	114284740	114284844	104
Exon 9	ENSE00003491906	114652203	114652290	88	114292258	114292345	87
Exon 10	ENSE00003652522	114653926	114654009	84	114293981	114294064	83
Exon 11	ENSE00003572969	114658066	114658267	202	114298121	114298322	201
Exon 12	ENSE00003585047	114659356	114659432	77	114299411	114299487	76
Exon 13	ENSE00003606163	114659572	114659673	102	114299627	114299728	101
Exon 14	ENSE00003527883	114662065	114662186	122	114302120	114302241	121
Exon 15	ENSE00003543277	114663450	114663519	70	114303505	114303574	69
Exon 16	ENSE00003498832	114664273	114664436	164	114304328	114304491	163
Exon 17	ENSE00003785442	114689782	114691745	1964	114329837	114329981	144

Table S2: primer sequences and PCR conditions for amplification of FOXP2 exons 4 – 17 using Sanger sequencing

Exon	Primer sequence (5' - 3')	Amplicon (bp)	T_M	Mg^{++}	Primer concentration (nM)	PCR cycles
Exon 4 fwd	acagatatttggttatgaccacg	242	56	1.5 mM	16 pmol	32
Exon 4 rev	gcacaccttctaagtcatgctag					
Exon 5-6 fwd	tttggctctgaccctttagaa	541	56	1.5 mM	16 pmol	32
Exon 5-6 rev	atctaagactattcttgccgctc					
Exon 7 fwd	caggcaatgaaaggagtgtgc	339	56	1.5 mM	16 pmol	32
Exon 7 rev	agaaaggccatgaaatggtag					
Exon 8 fwd	ctttaccttgttatgctagtg	335	56	1.5 mM	16 pmol	31
Exon 8 rev	attgttctttttcattgtctc					
Exon 9 fwd	tgctctgagctgaattgacc	330	52	1.5 mM	16 pmol	31
Exon 9 rev	gacttttacattcctccttcatc					
Exon 10 fwd	gtagtgctttttaagtgtagcc	230	56	1.5 mM	16 pmol	32
Exon 10 rev	tcagtactcaatgtaacacacc					
Exon 11 fwd	ggcaagctcaatgataagatgtatc	288	54	1.5 mM	16 pmol	32
Exon 11 rev	tattgccgtatttttcatcacactc					
Exon 12 fwd	gaatccactctcatttgtcaaacc	371	56	1.5 mM	16 pmol	32
Exon 12 rev	ctctggaatggctcatgtacag					
Exon 13-14 fwd	agaaattgccccaaactatga	367	56	1.5 mM	16 pmol	32
Exon 13-14 rev	ctgtaaggaactgtggtagg					
Exon 15 fwd	tagtatgttgggctgccttattag	230	54	1.5 mM	16 pmol	32
Exon 15 rev	gatgcaggatacaggattacaaaac					
Exon 16fwd	ctattagtgtgagacaagccag	218	56	1.5 mM	16 pmol	32
Exon 16 rev	gccctaaacattcaaatacaac					
Exon 17 fwd	ggaacccattaaaagaagatac	286	54	1.5 mM	16 pmol	32
Exon 17 rev	attcttccctttagagagtctg					
Exon 18 fwd	tcttcactgcaaagttggcc	395	54	1.5 mM	16 pmol	32
Exon 18 rev	ttgcctgttggtactgaatcc					

T_M = Annealing temperature, Mg^{++} = Magnesium concentration

Table S3: primer sequences and PCR conditions for amplification of poly Q microsatellites

**Microsatellite**	**Primer sequence (5' - 3')**	**Annealing Temperature (°C)**	**Primer concentration (nM)****	**PCR cycles**
Q1 exon 5 fwd*	AGA GCA GTT ACA TCT TCA GC	49	10	35[1]
Q1 exon 5 rev	ACC TCT TTC GCT TGC TTT C			
Q2 exon 6 fwd*	CTG CTG TTT ACT GGT TTG GGT TTT C	49	10	30[2]
Q2 exon 6 rev	CTG GAA GAC AAG CTG CTG GG			

***Forward primers were fluorescently labelled (Q1: 6-FAM; Q2: HEX). ****12.5 µL PCR reaction mix contained 6.25 µL QIA hotstar master mix (Qiagen), 0.5 µL of each primer, 3.25 µL of water and approximately 40ng of genomic DNA. [1] Initial incubation at 95°C (10 minutes), followed by 35 cycles at 95°C (30 s), 49°C (40 s), 72°C (40 s) and a final extension period of 10 minutes at 72°C. [2] PCR reaction mix and cycle conditions identical to poly Q1 except for number of cycles and the final extension period, which were set to 30 cycles and 30 minutes respectively.

Table S4: List of genome names and species included in dN/dS selection modelling

GENOME IS	GENUS	SPECIES	COMMON NAME
**AILMEL1**	*Ailuropoda*	*melanoleuca*	Giant panda
**BOSTAU7**	*Bos*	*taurus*	Cow
**CALJAC3**	*Callithrix*	*jacchus*	Common marmoset
**CAMFER1**	*Camelus*	*ferus*	Wild Bactrian camel
**CANFAM3**	*Canis*	*familiaris*	Domestic dog
**CAPHIR1**	*Capra*	*hircus*	Goat
**CAVPOR3**	*Cavia*	*porcellus*	Guinea pig
**CERSIM1**	*Ceratotherium*	*simum*	White rhinoceros
**CHILAN1**	*Chinchilla*	*lanigera*	Long-tailed chinchilla
**CHLSAB1**	*Chlorocebus*	*sabeus*	Green monkey
**CHRASI1**	*Chrysochloris*	*asiatica*	Cape golden mole
**CONCRI1**	*Condylura*	*cristata*	Star-nosed mole
**DASNOV3**	*Dasypus*	*novemcinctus*	Nine banded armadillo
**ECHTEL2**	*Echinops*	*telfairi*	Lesser hedgehog tenrec
**ELEEDW1**	*Elephantulus*	*edwardii*	Elephant shrew
**EPTFUS1**	*Eptesicus*	*fuscus*	Big brown bat
**EQUCAB2**	*Equus*	*caballus*	Horse
**ERIEUR2**	*Erinaceus*	*europaeus*	European hedgehog
**FELCAT5**	*Felis*	*catus*	Domestic cat
**GORGOR3**	*Gorilla*	*gorilla*	Gorilla
**HETGLA2**	*Heterocephalus*	*glaber*	Naked mole rat
**JACJAC1**	*Jaculus*	*jaculus*	Lesser Egyptian jerboa
**LEPWED1**	*Leptonychotes*	*weddellii*	Weddell seal
**LOXAFR3**	*Loxodonta*	*africana*	African elephant
**MACFAS5**	*Macaca*	*fascicularis*	Crab-eating macaque
**MESAUR1**	*Mesocricetus*	*auratus*	golden hamster
**MICOCH1**	*Microtus*	*ochrogaster*	Prairie vole
**MM10**	*Mus*	*musculus*	House mouse
**MUSFUR1**	*Mustela*	*putorius*	Ferret
**MYODAV1**	*Myotis*	*davidii*	David's bat
**MYOLUC2**	*Myotis*	*lucifugus*	Little brown bat
**NOMLEU3**	*Nomascus*	*leucogenys*	Northern white-cheeked gibbon
**OCHPRI3**	*Ochotona*	*princeps*	Pika
**OCTDEG1**	*Octodon*	*degus*	Common degu
**ODOROSDIV1**	*Odobenus*	*rosmarus*	Pacific walrus
**HG19**	*Homo*	*sapiens*	Human
**ORCORC1**	*Orcinus*	*orca*	Orca whale

**ORYAFE1**	*Orycteropus*	*afer*	Aardvark
**ORYCUN2**	*Oryctolagus*	*cuniculus*	European rabbit
**OTOGAR3**	*Otolemur*	*garnettii*	Northern greater galago
**OVIARI3**	*Ovis*	*aries*	Sheep
**PANHOD1**	*Pantholops*	*hodgsonii*	Tibetan antelope
**PANTRO4**	*Pan*	*troglodyetes*	Chimpanzee
**PAPHAM1**	*Papio*	*hamadryas*	Hamadryas baboon
**PONABE2**	*Pongo*	*abelii*	Sumatran orangutan
**PTEALE1**	*Pteropus*	*alecto*	Black flying fox
**PTEVAM1**	*Pteropus*	*vampyrus*	Large flying fox
**RHEMAC3**	*Macaca*	*mulatta*	Rhesus macaque
**RN5**	*Rattus*	*norvegicus*	Norway rat
**SAIBOL1**	*Saimiri*	*boliviensis*	Black-capped squirrel monkey
**SORARA2**	*Sorex*	*araneus*	European shrew
**SPETRI2**	*Spermophilus*	*tridecemlineatus*	Thirteen-lined ground squirrel
**SUSSCR3**	*Sus*	*scofa*	Pig
**TRIMAN1**	*Trichechus*	*manatus*	West Indian manatee
**TUPCHI1**	*Tupaia*	*chinensis*	Chinese treeshrew
**TURTRU2**	*Tursiops*	*truncatus*	Common bottlenose dolphin
**VICPAC2**	*Vicuna*	*pacos*	Alpaca

Supplementary Methods 1: Details on software and methodology used for dN/dS analysis

We downloaded the multiZ 100-way vertebrate alignment for the canonical transcript of *FOXP2* from the UCSC Genome Browser [1]. We analyzed the alignment using the Ete Toolkit (version 3.0.0b36), a python-based phylogenetic analysis package [2]. Within the Ete Toolkit, RAxML (version 8.1.20)[3] was first used to infer the gene tree from the alignment. Next, sitewise dN/dS was estimated and evidence for selection tested for within a maximum likelihood framework using the Sitewise Likelihood Ratio (SLR) method [4] and codeml site models [5]. SLR performs a likelihood ratio test (LRT) at each site in a protein to detect, after correcting for multiple tests, significant deviations from neutrality (purifying or positive selection) at a site. In codeml, a null model (M0) of a single dN/dS across sites is compared via LRT with a model of two classes of dN/dS (M1; purifying and neutral) and three classes of dN/dS (M2; purifying, neutral, and positive).

References used:

1. Kent, W. *et al.* The human genome browser at UCSC. *Genome Res.* **12,** 996–1006 (2002).
2. Huerta-Cepas, J., Serra, F. & Bork, P. ETE 3: reconstruction, analysis, and visualization of phylogenomic data. *Mol. Biol. Evol.* **33,** 1635–1638 (2016).
3. Stamatakis, A. RAxML version 8: a tool for phylogenetic analysis and post-analysis of large phylogenies. *Bioinformatics* **30,** 1312–1313 (2014).
4. Massingham, T. & Goldman, N. Detecting amino acid sites under positive selection and purifying selection. *Genetics* **169,** 1753–1762 (2005).
5. Yang, Z., Nielsen, R., Goldman, N. & Pedersen, A. Codon-substitution models for heterogeneous selection pressure at amino acid sites. *Genetics* **155,** 431–449 (2000).

Table S5: Single nucleotide variations for *FOXP2* coding sequences across hominoids. Coding variants are bolded and underlined. Single nucleotide polymorphisms are indicated following IUPAC ambiguity codes. Grey shading indicates variants located inside the variable poly Q stretches.

**DNA base location in human coding sequence**	17	136	180	249	258	270	354	381	390	414	441	459	462
human	C	A	A	A	G	A	A	T	G	G	G	A	A
Chimpanzee	C	**W**	G	R	G	A	C	T	G	G	G	A	A
Bonobo	C	A	A	A	R	A	C	T	G	G	R	A	A
Gorilla	C	A	A	A	G	G	C	T	K	R	G	R	R
Orangutan	**T**	A	A	A	G	A	C	C	G	G	G	R	A
Gibbon	C	A	A	A	G	A	C	C	G	G	G	G	A

**DNA base location in human coding sequence**	465	468	471	480	486	490	498	504	507	522	579	627	645
human	A	A	A	A	G	G	G	G	G	G	A	A	G
Chimpanzee	A	R	R	A	G	G	G	G	G	G	A	A	G
Bonobo	A	A	R	A	G	G	G	G	G	G	A	A	G
Gorilla	R	A	R	A	G	G	G	G	G	G	A	A	G
Orangutan	A	A	A	R	R	R	R	R	R	G	R	A	G
Gibbon	A	A	A	A	G	G	G	G	G	A	G	G	A

**DNA base location in human coding sequence**	654	702	860	873	900	926	928	1005	1068	1149	1416	1437	1494
human	C	A	**A**	A	A	**G**	G	A	A	C	A	T	G
Chimpanzee	C	A	C	G	C	A	G	M	A	C	R	T	G
Bonobo	M	A	C	G	C	A	G	A	A	C	A	T	G
Gorilla	C	A	C	A	A	A	**K**	A	R	C	A	T	A
Orangutan	C	C	C	A	A	A	G	A	A	Y	A	Y	G
Gibbon	C	A	C	A	A	A	G	A	A	C	A	T	G

**DNA base location in human coding sequence**	1563	1803	1831	1842	1878	1908	1932	1941	1968	1992	2016	2046
human	A	A	C	A	C	T	G	A	C	A	C	A
Chimpanzee	A	A	C	A	Y	C	G	A	C	A	M	A
Bonobo	A	A	C	A	C	C	G	A	C	A	C	A
Gorilla	A	R	C	A	C	T	G	A	T	A	C	G
Orangutan	G	A	**M**	C	C	T	G	R	T	A	C	A
Gibbon	A	A	C	C	C	T	A	G	T	G	C	A

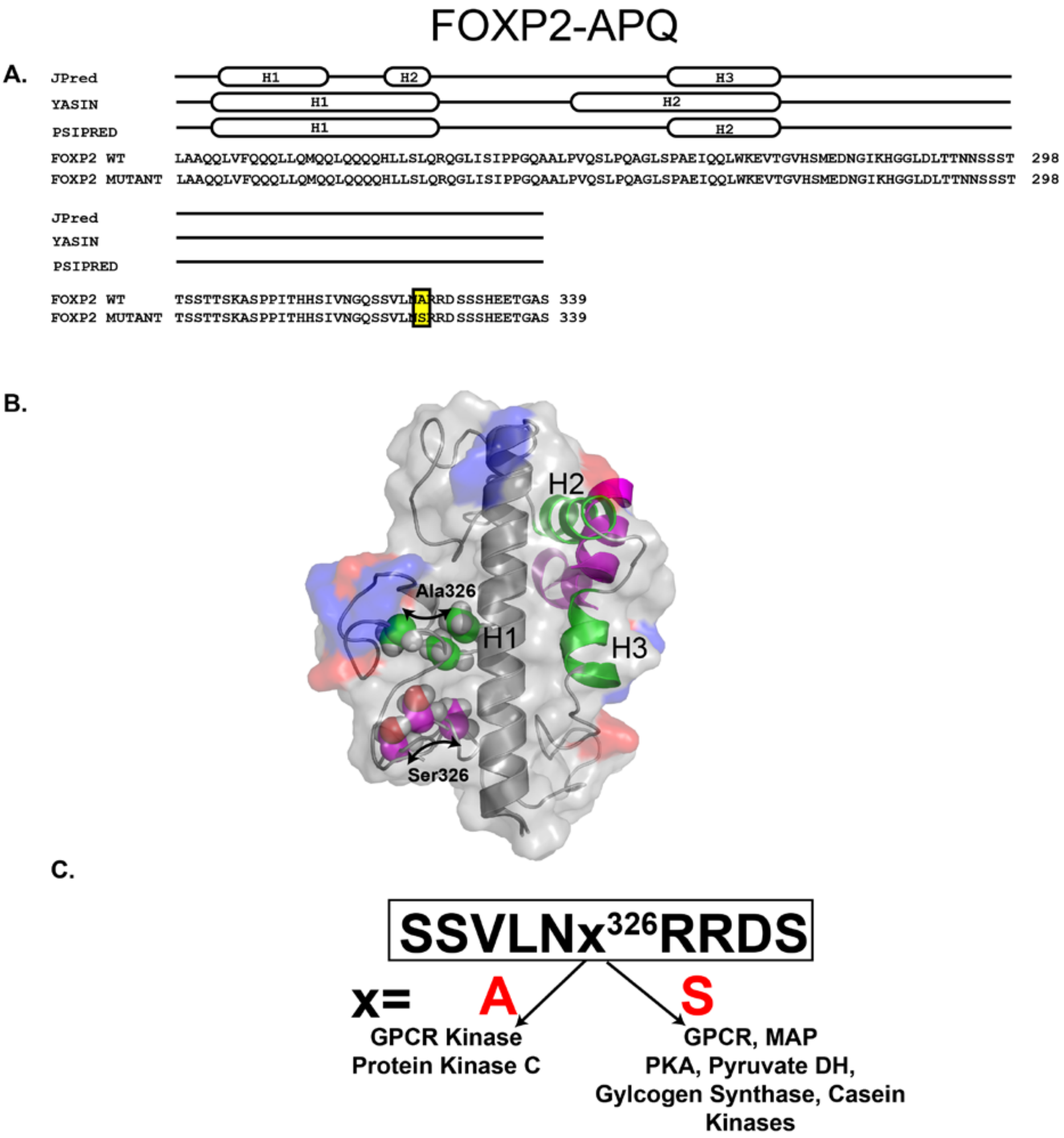

Figure S1: Structure prediction of FOXP2 N- and C-terminal regions. A. Sequence alignment of the wild-type and Ala326Ser mutant FOXP2-APQ region using three different algorithms. **B.** QUARK predicted structure of the FOXP2-APQ region. Structures of three wild-type and three mutant structures are superimposed. The central helix of one structure is shown while helix 2 and 3 for both wild-type (green) and mutant (magenta) structures are shown to indicate variations within each set of structures. Similarly, the variations in locations of Ala326 (green) and Ser326 (magenta) are shown in spheres. In addition, a surface representation of the structure depicting basic (blue) and acidic (red) residue distribution is depicted. **C.** Amino acid sequence of adjacent residues to the site of mutation showing potential kinase recognition motifs introduced by the Ser326 mutation.

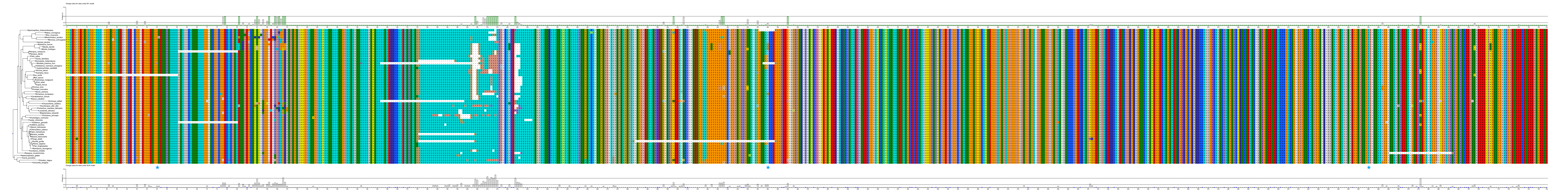

Supplement: Supplementary file 1 — Supplementary information [file 41598_2017_16844_MOESM1_ESM.pdf]
